# Low-dimensional metal chalcogenides for wearable gas sensing

**DOI:** 10.1186/s40580-025-00500-6

**Published:** 2025-07-10

**Authors:** Yanyan Li, Yuxiang Zhang, Haiyun Ma, Yi Wan, Tianshuo Zhao

**Affiliations:** 1 Department of Electrical and Electronic Engineering, The University of Hong Kong, Hong Kong, People’s Republic of China; 2 Materials Innovation Institute for Life Sciences and Energy (MILES), HKU-SIRI, Shenzhen, People’s Republic of China; 3 Department of Materials Science and Engineering, National University of Singapore, Singapore, 117575 Singapore

**Keywords:** Low-dimensional metal chalcogenides, Material surface design, Wearable gas sensors, Room temperature gas sensing, Machine learning assisted sensor arrays

## Abstract

Real-time monitoring of the surrounding gas environment, including our inhaled and exhaled atmosphere, is a crucial but underdeveloped technology for personalized healthcare. Recent advancements in wearable sensing technologies and AI algorithms promise the realization of more powerful wearable gas sensing systems, such as electronic noses. However, fundamental studies are still ongoing in seeking efficient gas sensing materials, transducing mechanisms, and device structures to meet the basic requirement of wearability and low power operation. Low-dimensional metal chalcogenides have attracted significant attention in building flexible gas sensors with room-temperature operation. Their controllable synthesis and post-synthesis treatment allow precise manipulation of the gas adsorption and charge transfer process. Their high surface-to-volume ratio, abundant active surface sites, and tunable electronic properties enable high sensitivity and selectivity, and fast response/recovery even without thermal activation. This review begins with an overview of three transducing mechanisms, providing a comprehensive understanding of the gas sensing process. Aiming at achieving efficient transducers, different types of low-dimensional metal chalcogenides, especially the 0D quantum dots and 2D nanosheets families, have been discussed regarding their synthesis methods and key material design strategies. State-of-the-art low-dimensional metal chalcogenide gas sensors are analyzed based on their modifications to the gas adsorption energy, charge transfer rate, and other fundamental parameters. Moreover, potential system construction towards smart and wearable gas sensor devices has been described with the integration of diversified sensor arrays, wireless communication technologies, and AI algorithms. Finally, we propose the remaining challenges and outlook for developing low-dimensional metal chalcogenide wearable gas sensing and eventually achieving accurate gas mixture classification and odor recognition.

## Graphical abstract

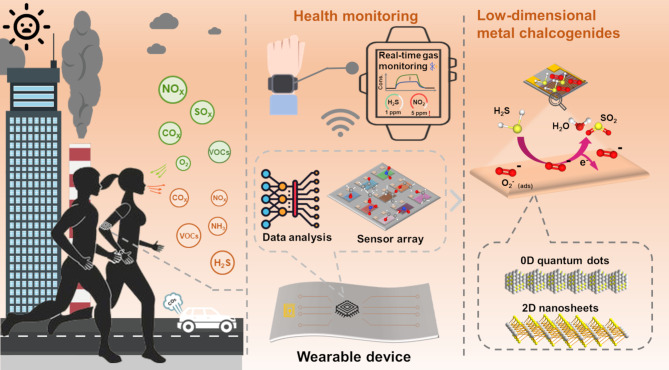

## Introduction

Gas molecules constantly interact with human bodies through breath, underscoring the critical role of analyzing both inhaled and exhaled gases for comprehensive health monitoring. For example, inhaling nitrogen dioxide (NO_2_) emitted through fossil fuel combustion can irritate lungs and induce respiratory infections when the concentration exceeds 3 ppm [1]. In the exhaled breath, trace amounts of volatile organic compounds (VOCs) can serve as biomarkers for various diseases and health conditions. The concentration of acetone in the exhaled breath of healthy individuals is typically below 1 ppm, while it exceeds 1.7 ppm and 2.2 ppm in patients with type 2 and type 1 diabetes, respectively [2, 3]. As a result, there is an urgent need for high-precision, real-time gas sensing technologies to monitor air quality, environmental pollutants, and physiological markers during our daily activities.

Gas sensors transform the concentration of target gases into measurable electrical [4, 5], optical [6], or acoustic signals [7], depending on their transducing mechanisms. The performance of gas sensors is primarily evaluated by several key metrics: sensitivity (the ratio between output signal change due to gas exposure and the original signal), limit of detection (LOD, the minimum gas concentration that can be detected), response time (the time required to reach at 90% of total response upon exposure to the gas) and recovery time (the time required to return to 10% of total response after the gas is removed), selectivity (the ability to distinguish the target gas from other interfering gases), and stability (retention of response after on/off cycles of the target gas) [8].

The development of wearable devices enables continuous and non-invasive monitoring of the surrounding gas environment. Wearable gas sensors facilitate early detection of potential health hazards, disease management, and personalized healthcare interventions. To improve wearability, gas sensor systems demand low-power operation, flexible and lightweight form factors, and wireless data transmission. However, there are still critical challenges to meet these requirements.

- Traditional gas sensors’ sensitivity and response/recovery time are unsatisfactory under low-temperature or room-temperature operation [9]. The most widely studied metal oxide gas sensors need to be heated to activate surface-adsorbed oxygen. The highly reactive oxygen species can accelerate the redox reaction kinetics with the target gas molecules at the gas-solid interface [10, 11]. The high operation temperature (> 200 °C) is the major contributor to the power consumption of the existing sensor device (5 mW) [12]. However, at lower or room temperature, the active oxygen radicals O_2_^-^ are less reactive, diminishing the sensitivity towards low-concentration gas. For other sensing materials with high surface adsorption energy at room temperature, gas molecules are easily adsorbed but difficult to desorb, resulting in prolonged or incomplete recovery [13]. Therefore, it is essential to design active sites or introduce auxiliary stimuli that can effectively modulate gas adsorption and desorption, in order to achieve both high sensitivity and rapid recovery at room temperature.
- The classification of multi-gas mixtures is limited by the selectivity of gas sensors [14]. Designing the surface chemistry to tune the adsorption energy is an essential way of obtaining higher affinity towards certain gas molecules. However, this method can be less effective for gases with similar molecular structures, compositions, and thus reactivity, such as some VOCs. For a fixed sensing material composition, the tunability might also be limited to cover a narrow range of gases. One promising upgrade is to form sensor arrays by including sensor devices with different selectivity [15, 16]. The complex sensory data matrix needs to be processed with efficient algorithms to distinguish gas mixtures [17]. However, if all measurements are based on the same transducing mechanism, the output signals from the sensor array may be too similar to provide a sufficient signature for specific gases. It is thus necessary to design and incorporate diversified transducers in the sensor array to increase the sensory data dimensionality and construct multi-layer algorithms with higher accuracy.
- Conventional gas sensor systems, including sensor chips, power sources, and data transmission/storage/processing units, are not designed for wearability. Battery packs are required to support the high power consumption of gas sensors and data computation. Also, bulky circuit components are placed next to the sensor to collect, transfer, and process the data. The entire system is typically with rigid and heavy packaging. Therefore, a holistic design of the sensor materials and device structures is critical to improve power efficiency and reduce the system weight. Flexible gas sensors are more advantageous for integration on diverse surfaces. Wireless data communication is to be developed for data processing and visualization with mobile device terminals, like smartphones.

To tackle these challenges, gas sensor systems, including the sensing materials, transducer mechanisms, and signal processing units, should be renovated. Metal chalcogenides, including transition metals (like Mo, W), group III and group IV metals (like In, Sn, and Pb), and chalcogenides (like S, Se, and Te), represent a promising class of materials for room-temperature gas sensing. Their low-dimensional structures, including 0D nanoparticles or quantum dots (QDs), 1D nanowires/nanorods, 2D nanosheets, and their nanocomposites, are particularly attractive for high surface reactivity with the large surface-to-volume ratio and abundant surface defects [18]. Their moderate bandgap energies enable various gas-sensing mechanisms at room temperature, while their precisely controlled synthesis and fabrication enable the integration with complex device array designs.

**Fig. 1:**
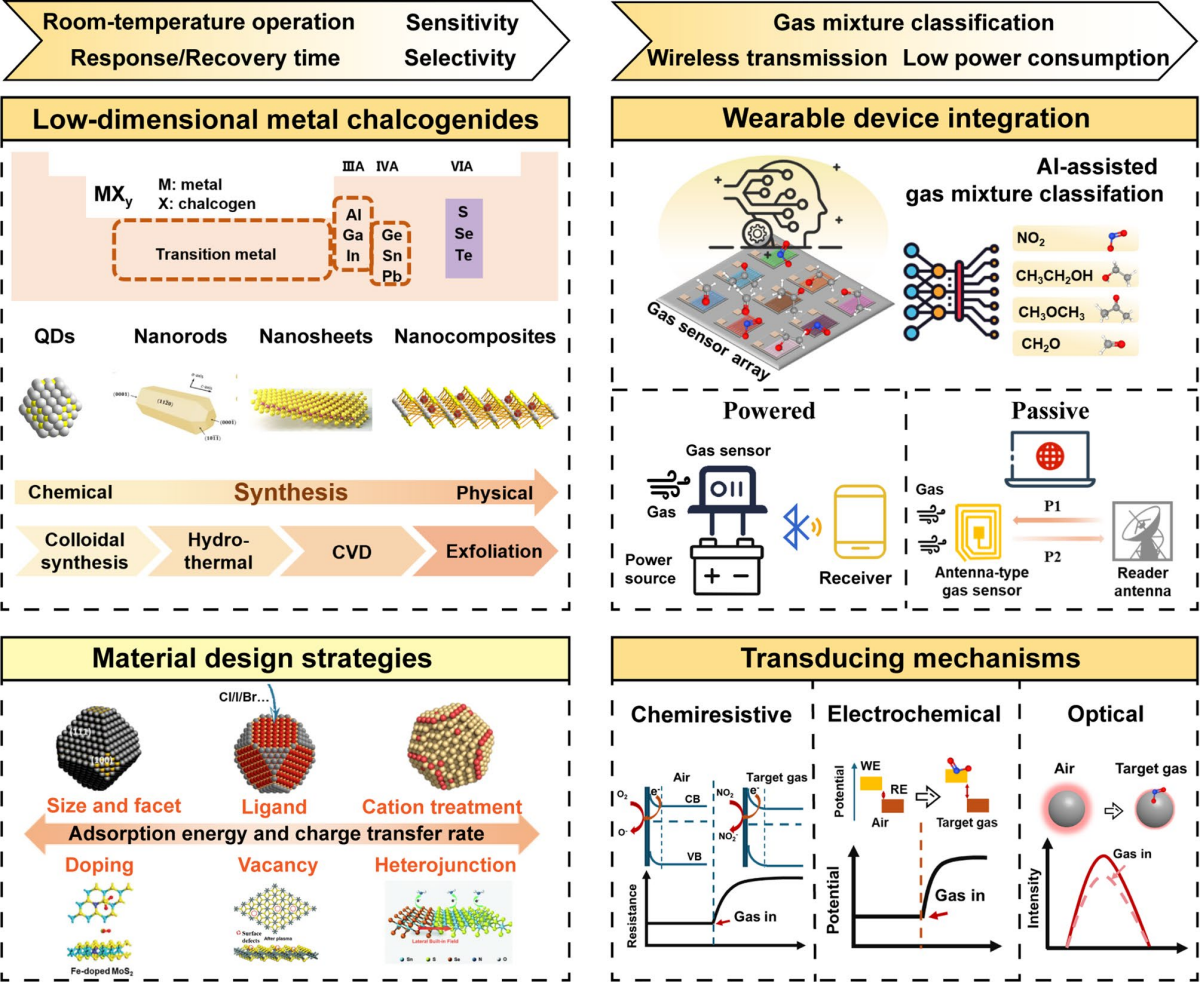
Critical aspects and outline of this review for achieving low-dimensional metal chalcogenide-based room-temperature wearable gas sensors

This article provides a focused review of recent advances in room-temperature wearable gas sensors based on low-dimensional metal chalcogenides (Fig. 1). First, we summarize three gas sensing mechanisms, namely chemiresistive, electrochemical, and optical sensing. They serve as fundamental guidelines for the design of sensing materials. Then, we introduce different types of low-dimensional metal chalcogenide systems and their material design strategies for gas sensing applications, especially emphasizing 0D QDs and 2D nanosheets as widely reported systems. The controllable synthesis enables their homogeneous and widely tunable compositions, sizes/thickness, surface sites, and hence electrical and optical properties. Both during and post-synthesis strategies have been developed for low-dimensional metal chalcogenides to carefully modulate their surface reactivity, gas adsorption energies, and charge transfer rates with the target gas. We exemplify these approaches that play critical roles in achieving high sensitivity, fast response and recovery, high selectivity, and other gas sensor metrics. Finally, we explore potential system integration methods to achieve fully wearable gas sensor devices and electronic nose applications. State-of-the-art systems demonstrate low-power and no-power (passive) operations, wireless data communication, and gas classification based on sensor arrays and data processing algorithms. This recent progress also leads to our perspectives on future directions to overcome the remaining challenges in developing low-dimensional metal chalcogenide-based wearable gas sensors.

## Transducing mechanisms

The adsorption of gas molecules causes alterations in the electrical and optical properties of the transducing materials, which are converted to measurable parameters of the sensor devices. This review discusses three transducing mechanisms: chemiresistive, electrochemical, and optical gas sensing.

### Chemiresistive gas sensing

**Fig. 2:**
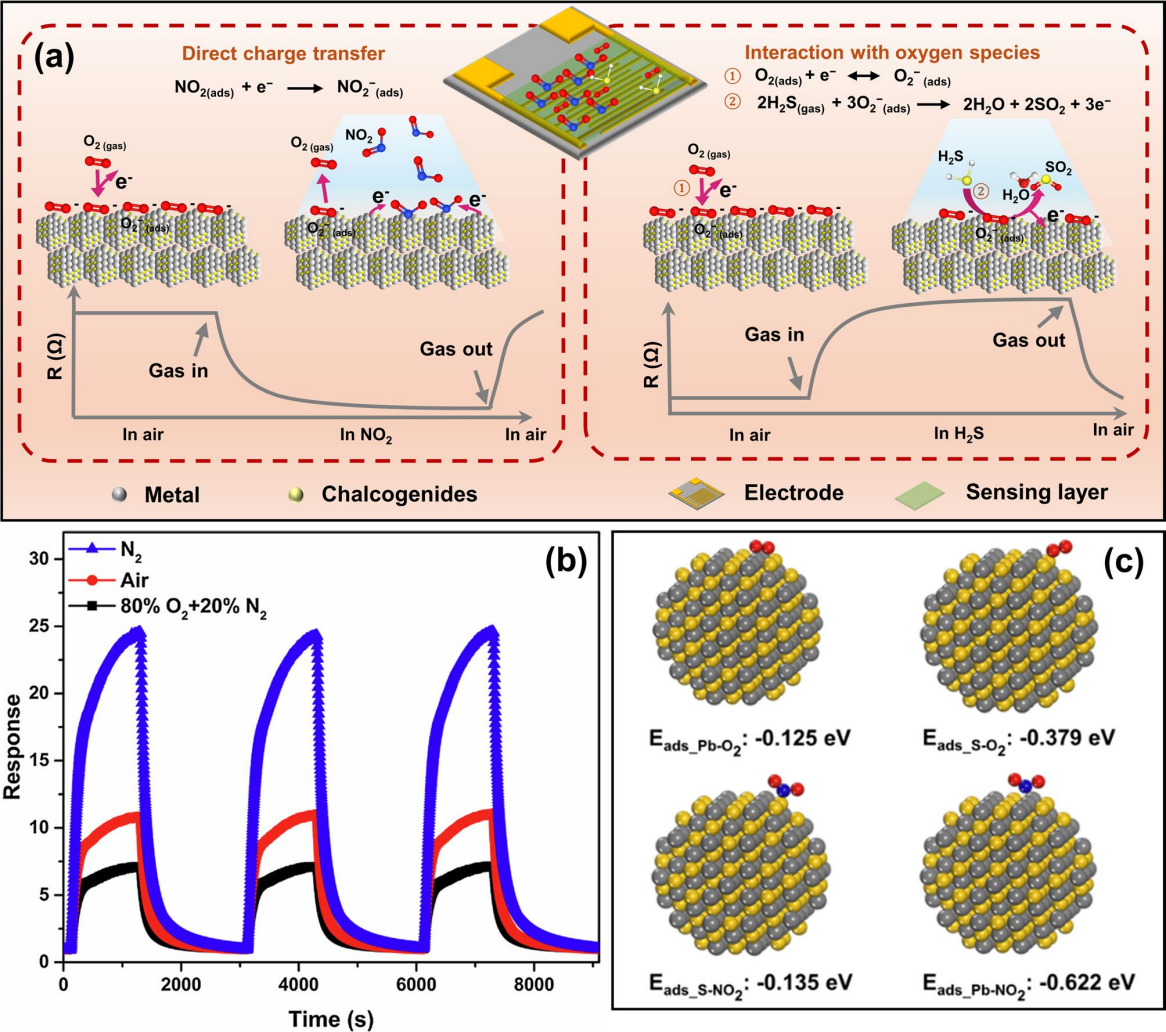
Gas sensing mechanisms of chemiresistive low-dimensional metal chalcogenide gas sensors. (**a**) Schematics of a p-type sensing layer interacting with NO_2_ molecules through direct charge transfer (left) and with H_2_S molecules through adsorbed oxygen-assisted charge transfer (right), and corresponding response curves (the opposite trend of resistance change for n-type sensing materials). (**b**) Response curves of the SnS_2_ nanosheet sensor to 500 ppb of NO_2_ tested in N_2_ (blue), air (red), and 80% O_2_ + 20% N_2_ (black) background, respectively [28]. (**c**) The adsorption energy of O_2_ (top) and NO_2_ (bottom) binding to surface Pb and S sites of PbS QDs [29]

Chemiresistive gas sensors are typically two-terminal devices, where the electrodes are in electrical contact with the sensing layer to measure the resistance change induced by target gas exposure. The gas response is defined as $\:\left|{R}_{g}-{R}_{a}\right|/{R}_{a}\:\times\:100\%$, where $\:{R}_{a}$ refers to baseline resistance under dry air, $\:{R}_{g}$ refers to resistance under target gas exposure. The response of metal oxide-based gas sensors typically requires thermal activation by integrated microheaters, but low-dimensional metal chalcogenide devices can exhibit room-temperature gas response. The latter is explained in two basic models as shown in Fig. 2 [19, 20].

**DIRECT CHARGE TRANSFER.** When a target gas molecule is adsorbed on metal chalcogenides, it can undergo redox reactions by drawing or donating electrons from the sensing material. Consequently, the carrier concentration of the sensing layer is directly altered, leading to measurable changes in resistance or output current under a constant applied voltage. Upon complete gas desorption, the sensing material returns to its initial state, and the resistance recovers to the baseline. Oxidizing gases (e.g., NO_2_, NO, SO_2_) with high electron affinity act as electron acceptors, and their reduction reactions typically have more positive redox potentials than the conduction band edge of the sensing material. Therefore, electrons are drawn from metal chalcogenides to the adsorbed oxidizing gas molecules, resulting in a decrease in electron concentration of the sensing material and reduction of the gas molecules. For n-type semiconductors, where electrons are the majority charge carriers, exposure to oxidizing gases results in an increase in resistance [21]. P-type sensing materials, on the other hand, show a decreased resistance when interacting with oxidizing gases (Fig. 2a, left). Gases (e.g., NH_3_, H_2_S, acetone) with lower electron affinity or those that tend to lose electrons are known as reducing gases. They become oxidized and donate electrons to the sensing materials [22]. Therefore, n-type sensing material becomes less resistive with increased electron concentration, while p-type materials exhibit higher resistivity when detecting the reducing gas molecules.

**CHARGE TRANSFER THROUGH ADSORBED OXYGEN.** Another model proposes that the target gas reacts with active oxygen species adsorbed on the surface, resulting in electron transfer to or from the low-dimensional metal chalcogenides. Upon exposure to the dry air, O_2_ adsorbs onto the surface of metal chalcogenides and is reduced to O_2_^−^, leading to an increase or decrease in baseline resistance for n-type or p-type metal chalcogenide materials (Fig. 2a, right) [23]. The activated O_2_^−^ species bind with the target gas rapidly, further releasing electrons to or withdrawing electrons from the sensing material, depending on the relative redox potentials of target gases. The resultant resistance change follows the same trend as the direct charge transfer mechanism explained above. Wu et al. proposed that adsorbed oxygen species facilitate reducing gas sensing [24]. The pre-adsorbed oxygen species enhance the electron affinity of the metal chalcogenide surface, thereby promoting the adsorption of reducing gases. For example, H_2_S may react with the O_2_^−^ on the surface and release electrons to SnS_2_, leading to decreasing resistance [25]. It is potentially beneficial to design the metal chalcogenide sensing material for hosting more adsorption of active oxygen species. For example, the abundant dangling bond of SnO_2_ QDs/MoS_2_ nanocomposites provides more absorption sites for absorbed O_2_ molecules, exhibiting high activity in the sensing reaction with NH_3_ [26].

Nevertheless, the pre-adsorbed oxygen species are also found to compete with certain gases with high electron affinity, such as NO_2_, during surface reactions [27]. For example, Tang et al. observed that SnS_2_ nanosheets had a lower response to NO_2_ in an O_2_-containing atmosphere compared to the same device measured under an N_2_ atmosphere (Fig. 2b) [28]. To ensure that the target gas adsorption and direct charge transfer are thermodynamically favorable, the adsorption energy between the target gas and metal chalcogenide surface sites should be sufficiently high. Liu et al. calculated the adsorption energies between PbS QDs and NO_2_ using the Density Functional Theory (DFT) [29], which is shown to be higher than that between PbS QDs and O_2_ (Fig. 2c). Higher adsorption energy can result in enhanced sensitivity. However, it may also hinder target gas desorption and delay the recovery process. Therefore, a trade-off would be reached with rational design of the adsorption energy.

The dominant sensing mechanism, through direct charge transfer or adsorbed oxygen-assisted transfer, needs to be considered comprehensively over a variety of factors. Different types of gases varied in electron affinities can have preferred sensing routes. As discussed above, target gases with high electron affinity (NO_2_, NO, SO_2_) are prone to release electrons to sensing materials directly [30, 31]. And reducing gases (H_2_S, NH_3_, and VOCs) typically are thermodynamically driven to transfer charge carriers through pre-adsorbed O_2_^−^ [32]. Besides, the vacancies, dangling bonds, and specific facets that prevail in low-dimensional metal chalcogenides can also alter the adsorption energy and band alignment to effectively affect charge transfer routes and rates [26, 33–34, 35]. Humidity is another factor, where pre-adsorbed H_2_O molecules are dissociated to H^+^ and OH^−^ and tune the surface properties of metal chalcogenides [36]. They can directly participate in the NH_3_ sensing process by forming NH_4_^+^ ions, which further facilitate electron transfer with the pre-adsorbed O_2_^−^ species [37].

### Electrochemical gas sensing

**Fig. 3:**
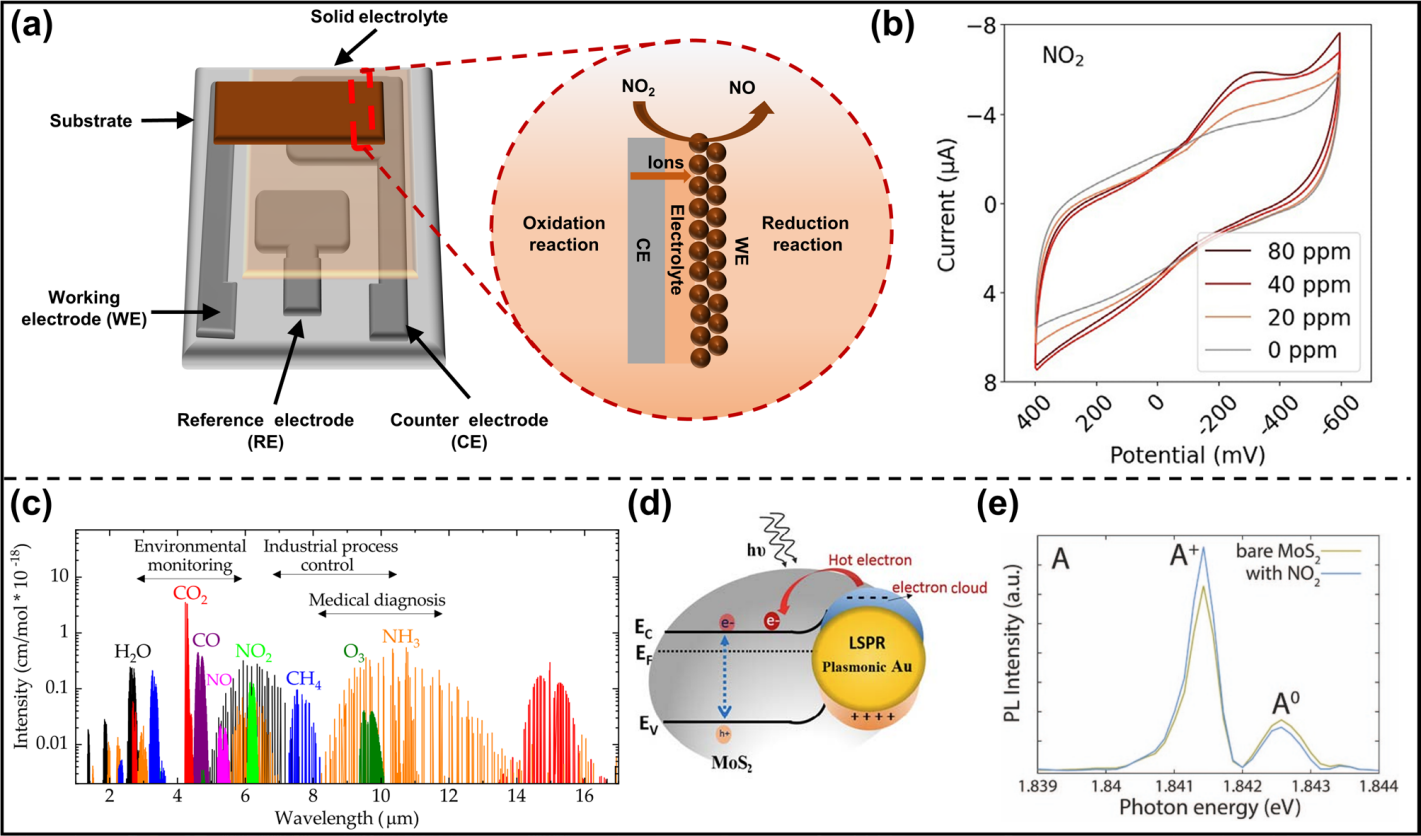
Mechanisms of electrochemical and optical gas sensing. (**a**) Schematics of general electrochemical gas sensing electrodes and mechanisms. (**b**) Cyclic-voltammetry measurements of NO_2_ mixed with the synthetic air at different concentrations [41]. (**c**) Mid-infrared absorption spectra of selected gas molecules [42]. (**d**) Schematic band energy diagrams of plasmonic Au nanoparticles and MoS_2_ nanosheets [47]. (**e**) In situ PL spectra of 2D MoS_2_ with (blue) and without (orange) NO_2_ gas molecules [22]

An electrochemical gas sensor typically consists of a working electrode (WE), a reference electrode (RE), a counter electrode (CE), and electrolytes (Fig. 3a) [38]. Such a three-electrode setup can characterize the chemical potential change of the sensing material induced by the redox reaction with the target gas that occurs at the gas-WE-electrolyte three-phase interface [39]. Based on the measured electrical signals, electrochemical sensors can be categorized into four types: potentiometric, amperometric, impedimetric, and conductometric sensors. The definition of gas response for the electrochemical gas sensor is similar to that of a chemiresistive gas sensor, which is described as the ratio between the change of electrical signal in the target gas environment and the initial electrical signal measured in the air.

Gas adsorption/desorption and associated charge transfer modify the chemical potential of the sensing material. As the WE, the sensing layer’s potential is changed relative to the RE potential that remains constant under controlled experimental conditions and is defined by a stable redox couple. The potential difference drives charge flows between the WE and the CE, which can be quantified by measuring various types of electrical signals. The correlation between electrical signals and gas concentration is utilized to calibrate the sensor device [40]. High sensitivity can be obtained by improving the conductivity and the number of active sites on the WE surface. Different redox reaction potentials of gas molecules give rise to distinctive patterns of their electrical parameter changes, which can be used to identify the gas species (Fig. 3b) [41]. Electrochemical gas sensing can operate with low power consumption and hence has the potential for portable devices. However, the range of detectable gas species using this method is limited. Its structure with multi-electrodes and liquid electrolytes is complicated. Metal chalcogenides, in particular, are more likely to suffer from instability after multiple redox cycles in liquid electrolyte environments [8].

### Optical gas sensing

The interaction between gas molecules and sensing materials can also lead to changes in optical properties, such as light absorption, scattering, and luminescence wavelengths and intensities. Such correlation has been used to quantify gas molecule concentrations via optical gas sensors.

Different gas molecules have characteristic absorption in the infrared (IR) region, corresponding to their molecular vibration or electronic transition energies (Fig. 3c) [42]. Therefore, the gas environment can be sensed by measuring the transmitted light spectra in this wavelength range [43, 44]. Broadband and miniaturized IR photodetectors are a critical enabling technology. Metal chalcogenide QDs, like PbS and HgTe QDs, are one of the major IR semiconductor families for high-efficiency and low-cost IR photodetectors, promising for optical gas sensing [45, 46].

Surface and localized surface plasmon resonance occur at the metal (usually a noble metal like gold or silver)/dielectric interface, causing strong scattering of light at the resonance wavelength. Such plasmonic resonance supports gas sensing, as the resonant wavelength and intensity are highly sensitive to the dielectric surrounding altered by the adsorption/desorption of gas molecules. Plasmonic gas sensors can be realized by loading metal nanoparticles or plasmonic nanostructures on low-dimensional metal chalcogenide devices. The presence of gas molecules on the surface of the Au nanoparticle/MoS_2_ nanosheets can affect the electron transfer processes and hence the photoresponse of the device (Fig. 3d) [47]. Some low-dimensional metal chalcogenides can also exhibit plasmonic resonances. For example, taking advantage of the precise doping and morphology control, Cu_x_S nanocrystals are heavily doped and subwavelength in size to support a high density of free carriers [48]. Their plasmonic properties can be sensitive to surface reactions with gas molecules.

Another widely reported method is to observe the photoluminescence (PL) intensity of metal chalcogenides under gas exposure [49, 50]. It can be quenched or enhanced through various mechanisms [51]. Metal chalcogenide QDs, such as CdSe QDs, are excellent light emitters. When gas molecules are adsorbed on the QD surface, the QD surface charge density and potential may be changed. Consequently, the QD energy levels and associated PL emission are distorted [52]. In addition, some gas molecules can passivate or create surface defects, reducing or increasing non-radiative recombination pathways to cause enhanced or weakened PL of metal chalcogenides. For 2D transition metal dichalcogenides (TMDs) like MoS₂ and WS₂, the PL quenching or enhancement upon gas adsorption is closely related to the charge transfer process. Electrons are readily transferred from the conduction band of 2D TMDs, such as n-type MoS₂, to the adsorbed gas molecules with high electron affinity, such as NO₂. Hence, the PL corresponding to the trion exciton A^+^ is reduced, but the neutral exciton A^0^ from a lower energy transition is enhanced (Fig. 3e) [22].

## Low-dimensional metal chalcogenides for gas sensing

### 0D metal chalcogenides − quantum dots

**Fig. 4:**
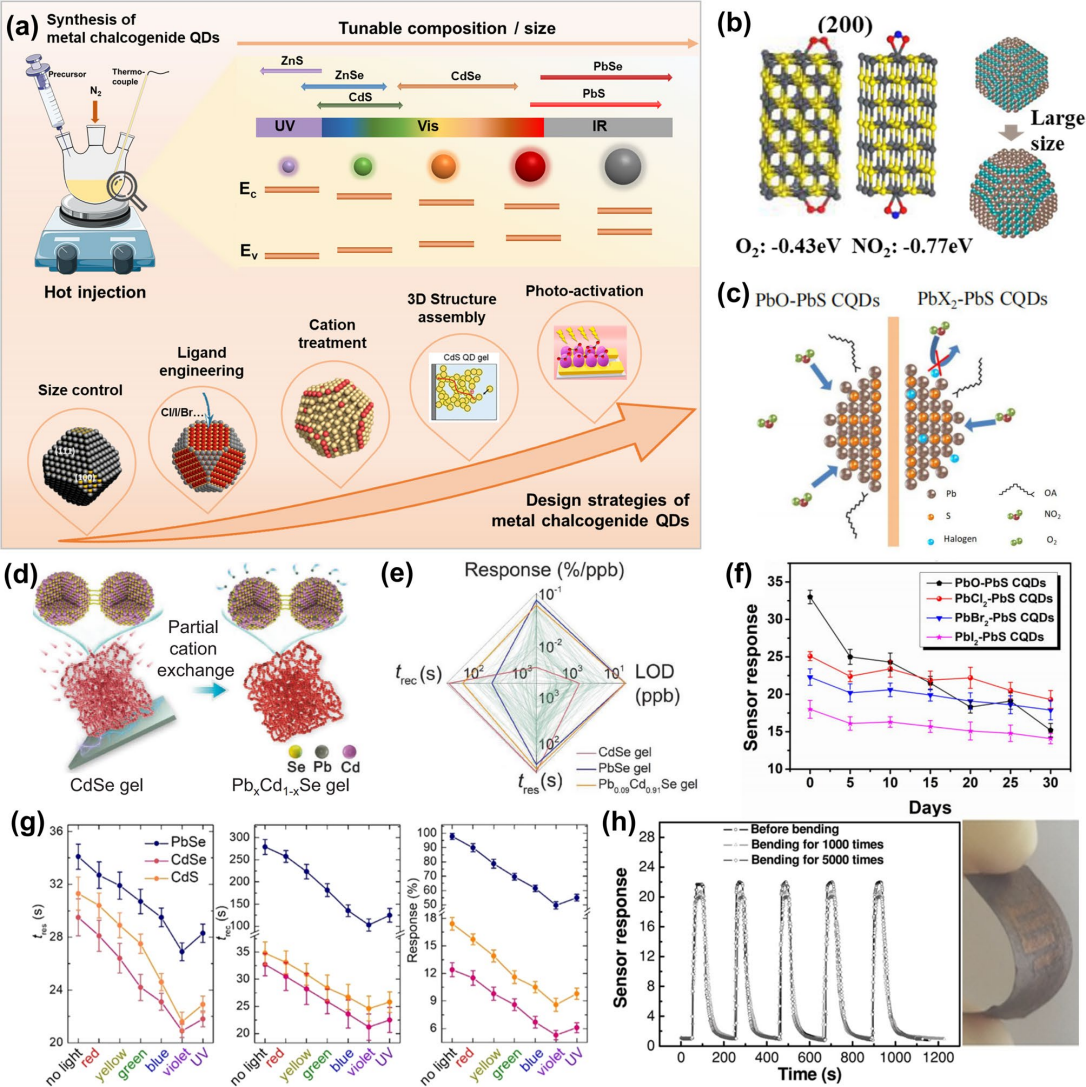
Colloidal metal chalcogenide QDs for gas sensing. (**a**) Colloidal synthesis and composition- and size-tunable metal chalcogenide QDs and design strategies for QD-based gas sensors. (**b**) The adsorption energies of O_2_ and NO_2_ on the (200) facet of PbS QDs (left) and the simulated crystal structures of different-sized PbS QDs (right) [77]. (**c**) Schematics of the gas adsorption on PbO–PbS QDs and PbX_2_–PbS QDs [79]. (**d**) Schematics of the synthesis of Pb_x_Cd_1−x_Se QD gels via partial cation exchange [80]. (**e**) Comparison of LOD, response, response time (t_res_), and recovery time (t_rec_) between CdSe, PbSe, Pb_0.09_Cd_0.91_Se QD gels, and other 110 room-temperature p-type NO_2_ gas sensors reported in the literature [80]. (**f**) Long-term stability of the PbS QD sensors synthesized with PbO (black), PbCl_2_ (red), PbBr_2_ (blue), and PbI_2_ (pink) [79]. (**g**) Response time (t_res_), recovery time (t_rec_), and gas response of PbSe (blue), CdSe (pink), and CdS (yellow) QD gel sensors illuminated by light with different wavelengths [86]. (**h**) Response curves of the PbS QD sensor before and after bending 1000 and 5000 times (left) and a photo of the flexible QD gas sensor (right) [19]

Quantum dots (QDs) are considered zero-dimensional nanomaterials. They consist of nanoscale semiconductor cores, typically smaller than 20 nm in diameter, which are comparable to exciton Bohr radii, and thus exhibit strong quantum confinement effects [53, 54]. The nanocrystalline cores are typically stabilized by capping ligands, including long-chain/short organic/inorganic molecules/ions. The extremely small dimension leads to high surface-to-volume ratios, supporting a large number of atoms and vacancies that can be converted to high-reactivity surface sites. By precisely controlling the particle size, QDs’ electronic band structures can be modulated, effectively regulating their optical and electrical properties **(**Fig. 4a**)**. The tunable energy band positions provide a facile approach to aligning QD energetics with the redox potentials of various gas molecules. The QD size can also vary the distribution of terminating crystal facets, engineering the surface sites to influence the adsorption and desorption behavior of target gas molecules [55].

Among the QD family, metal chalcogenide QDs have been considered efficient active materials in gas sensing applications. Compared to the most widely studied metal oxides, metal chalcogenide QD gas sensors can operate at lower temperatures. The major reason is the abundant active sites for gas adsorption induced by chalcogen vacancy, metal vacancy, the presence of dangling bonds, and capping ligands [56]. Direct and/or indirect charge transfer can occur between gas molecules and QDs without thermal excitation. Their narrower and tunable bandgap energies enable the photogeneration of carriers under UV-Visible to IR illumination wavelengths to activate/deactivate gas sensing. Another advantage of metal chalcogenide QDs, in contrast to metal oxides, is the ease of synthesis, post-treatment, and fabrication. Lower-temperature batch chemical synthesis is well-developed to prepare monodisperse QD dispersions, which are compatible with large-area solution-based processing. The device fabrication is typically free of aggressive annealing or sintering processes, making the QDs more suitable for flexible substrate integration.

#### Synthesis of metal chalcogenide QDs

In this review, we focus on colloidal QDs with their solution-based processing. For metal chalcogenide thin films grown by other methods, like liquid-phase epitaxy (LPE), metal-organic chemical vapor deposition (MOCVD), and molecular beam epitaxy (MBE), Galstyan et al. have systematically reviewed their synthesis and properties [57]. Colloidal metal chalcogenide QDs can be synthesized using a variety of chemical synthesis methods, including hot injection [58], solution precipitation [59], hydro/solvothermal [60], sol-gel [61], and thermal decomposition methods [62]. The hot injection, solution precipitation, and hydrothermal approaches are most widely adopted for preparing QD-based gas sensors, characterized by their simplicity, low reaction temperatures, and high tunability.

The hot injection method involves the rapid injection of low-temperature precursor solutions into high-temperature reaction solutions, leveraging the instantaneous temperature and concentration change to achieve controlled nucleation and growth of QDs [63]. As described by the LaMer model, upon injection, the precursors or converted monomers reach a supersaturated state within a short period, inducing burst nucleation [64]. As the nucleation process depletes monomer concentration below the supersaturation threshold, the QD growth dominates and may be accompanied by the Ostwald ripening process after extended growth times. Typically, for the synthesis of metal chalcogenide QDs, such as CdX (X = S, Se, Te) [65], PbX (X = S, Se, Te) [66], ZnX (X = S, Se) [65], and Cu_x_S [65], the metal precursor is first dissolved in a solution of ligands and coordinating or non-coordinating solvents and heated to a specific temperature. At this temperature, cold chalcogen precursor solutions are rapidly injected into the flask to initiate rapid nucleation. After a certain growth time, the reaction is quenched by quickly cooling down the solution. The reaction crude is subsequently purified by precipitating and redispersing QDs with anti-solvents and good solvents, respectively, to achieve the QD product dispersed in non-polar organic solvents. It is important to note that long-chain organic ligands play a crucial role during chemical synthesis by modulating the reactivity of precursors, stabilizing the QD surfaces, and narrowing the size distribution [67]. Therefore, by varying the concentrations of precursors and ligands, as well as modifying the reaction temperature and time, it is possible to precisely control the size, shape, and composition of QDs. This, in turn, allows for the fine-tuning of their surface properties and band structures.

The solution precipitation method has been reported to synthesize CdX (X = S, Se, Te), ZnX (X = S, Se), CuX (X = S, Se), and HgTe QDs [68–70]. The metal cation precursor solution is slowly injected into an aqueous or non-aqueous solution containing chalcogenide precursors under inert gas protection. QDs with controlled sizes are produced by tuning the reaction equilibrium between the nanocrystal growth and ion solvation. The reaction equilibrium is mainly affected by the precursor concentration, solvent type, pH, reaction temperature, and reaction time. The addition of capping agents can further modulate the particle size and dispersity of the QDs. QDs synthesized in the aqueous environment have poorer crystallinity than those synthesized in organometallic systems, due to the comparably low synthesis temperature [68]. Non-effective separation of nucleation and growth stages of precipitation approach leads to a broad size distribution of QDs [68].

The hydro/solvothermal method is a synthesis technique that utilizes water or organic solvents to host the reaction between precursors in a sealed high-pressure autoclave under elevated temperature and pressure [60, 71]. The high-temperature and pressured environment promotes the dissolution and conversion of precursors, forming a supersaturated solution that induces the nucleation and growth of QDs. Thanks to the uniform and enclosed reaction environment, QDs produced by the hydrothermal method typically exhibit high crystallinity. Similarly, the size and morphology of QDs can be controlled by adjusting the type of solvents, reaction temperature, reaction time, and the choice of surfactants. The hydrothermal method is more commonly adopted for preparing QDs in aqueous solutions, but their dispersibility is generally poor.

Among the three synthesis routes above, hot-injection yields QDs with high crystallinity, narrow size distribution, and excellent dispersity, which are necessary properties for constructing gas sensor devices. Nanocrystalline QDs exhibit more predictable physical properties, including higher carrier mobility and surface-active sites on certain facets [72, 73]. Fine-tuning of QD sizes reduces sample-to-sample and batch-to-batch variation, improving the reliability and reproducibility of QD gas sensors. The colloidal stability of QDs enables solution-based processing to fabricate and modify QD thin films. For example, post-synthesis solution-phase and solid-state ligand exchange treatments can homogenously tailor the QD properties in the dispersion and in the thin films, respectively, through engineering the surface chemistry [74]. Large-area and homogeneous thin films can be formed by spin/dip coating, spraying, printing, and blading QD solutions on various substrates [75].

#### Design strategies for metal chalcogenide QDs

The wide tunability of metal chalcogenide QDs’ physical and chemical properties promises desirable gas-sensing performance, including room-temperature operation, high sensitivity, and fast response/recovery time. These gas sensing metrics are determined by two critical steps, i.e., adsorption/desorption of gas molecules on/from the QD surface and charge transfer rates between the QD and gas molecules. Various strategies have been reported to improve these two aspects, essentially by designing the QD surface sites and electronic properties.

**QD SIZE CONTROL.** The surface stoichiometry of QDs, or the metal: chalcogen atomic ratio, plays a determining role in gas adsorption. Gas molecules prefer attaching to the surface metal or chalcogen atoms driven by the higher adsorption energy. Therefore, the terminating surface facets exposing different metal: chalcogen sites can be designed directly through QD size control. For example, as PbS QDs increase in size, more surface area is covered by (100) and (110) facets and less by (111) facets [76]. The former two have Pb: S ratios close to unity, while the (111) facet is mostly terminated by Pb atoms. Liu et al. synthesized PbS QDs with different sizes by changing the injection temperature and cooling process [77]. Through cold bath cooling, PbS QDs are synthesized with a size of 6.1 nm, exposing more (100) facets and exhibiting the optimal gas response to NO₂. DFT simulation shows that (100) facets facilitate the adsorption of NO₂ as a competitive pathway over oxygen adsorption on the PbS QD surface, as shown in Fig. 4b. In the meantime, the particle size also determines QD electronic properties, like the band structure, carrier concentration, and mobility of the QD thin film. These parameters also influence the interaction between QDs and the target gases with different redox potentials. Therefore, the effect of the QD size on gas sensing performance needs to be considered from the overall aspects in structural and electronic property modifications.

**LIGAND ENGINEERING.** Designing the QD surface with suitable ligands can alter the active site distribution, surface defects, charge transfer rate, and even band structures of the QD film. As-synthesized QDs are capped with long-chain ligands to retain colloidal stability. However, the long and insulating organic ligand shells block gas molecules from accessing active surface sites and impede the charge transport across the QD film. Therefore, compact ligands are introduced to replace the long ligands by ligand exchange treatment [78]. Specific ligand moieties have been studied to further passivate the QD surface and regulate the surface reaction with oxygen and other target gas molecules. Luo et al. synthesized PbS QDs, namely PbO-PbS and PbX_2_-PbS QDs, using lead oxide and lead halide as precursors, respectively [79]. PbX_2_-PbS QDs show more stable NO_2_ gas sensing performance than PbO-PbS QDs, owing to the surface halogen passivation effect **(**Fig. 4c and f**)**. However, another research also demonstrated that excessive halogen ligands can cover a large number of active sites and hinder charge transfer between the sensing material and NO_2_, significantly reducing the sensor response [29]. Metal salt solutions can also be used as ligand exchange reagents to provide metal cations in addition to inorganic anions to modify both types of surface sites simultaneously. This cation treatment process may also lead to cation exchange reactions to form alloy metal chalcogenide QDs and change the doping concentration and energy band positions. Luo et al. developed bimetallic Pb_x_Cd_1−x_Se QDs using partial cation exchange, where partial Cd sites were replaced by Pb, to obtain high gas sensitivity of 0.06% ppb^− 1^, a response time of ~ 28 s, and a recovery time of ~ 60 s (Fig. 4d and e) [80]. In comparison, pristine CdSe QDs show a low gas response, whereas PbSe QDs suffer from long response/recovery time. This cation exchange method introduces atomically dispersed Pb sites among the neighbouring Cd atoms, fine-tuning the adsorption strength toward NO_2_. Designing such bi-metallic surfaces could be an effective strategy to engineer the gas adsorption energy and charge transfer rate in achieving a high and rapid gas response.

**3D STRUCTURE ASSEMBLY.** QDs can be building blocks to assemble 3D porous structures to further increase the effective surface area and accessible active sites. Besides improving sensitivity, thin and porous 3D QD structures are also expected to promote the gas response and recovery process for enhanced diffusion of the target gas [19]. Spray-coated PbS QD films possess a more porous morphology than spin-coated QD films [81]. The NO_2_ gas response of the spray-coated porous PbS QD films is 2.6 times greater than that of the spin-coated and densely packed QD film, also achieving a short response time of 1 s and a recovery time of 14 s. Another work by Luo et al. demonstrates 3D-connected nanoarchitectures by electrochemical gelation of metal chalcogenide QDs (CdS, ZnS, and CdSe) with macroscale pores [82]. The process of gelation strips the organic ligands from the surface of QDs, creating more active sites. The porous structure still maintains the quantum confinement effect of individual QDs and ensures charge transport, yielding an ultralow LOD of 11 ppb and response/recovery time shorter than 30 s. The mechanism and more details of the gelation method are elaborated in the review paper by Luo et al. [83].

**PHOTO-ACTIVATION.** The wide tunability in size and composition of metal chalcogenide QDs offers bandgap energies ranging from UV to IR. The strong light-matter interaction suggests the feasibility of photo-activating QD gas sensors, where photo-generated carriers within the QDs interplay with the redox reaction of the target gas [84]. Also, as described by the Langmuir-Hinshelwood model, the wavelength and intensity of light irradiation significantly affect the photoactivated surface gas adsorption and desorption processes [85]. Tan et al. reported that illumination with wavelengths ranging from UV to red LED lights on QD films can shorten the gas response and recovery times, as shown in Fig. 4g [86]. This effect is attributed to the photoexcitation of free and adsorbed NO_2_ molecules. Specifically, NO_2_ gas molecules are photoexcited into a highly oxidative state, NO_2gas_*, leading to fast chemisorption kinetics and quick gas response. Meanwhile, the desorption rate of NO_2ads_ is enhanced under light illumination, due to the weakened adsorption energy between the adsorbed NO_2ads_ and the QD surface. Moreover, surface adsorbed O_2_ can be photoactivated to highly reactive O_2_^−^ (*hv*), which strongly interacts with reducing gases [87].

However, a large amount of photocarriers can be generated under illumination due to the high quantum efficiency of metal chalcogenide QDs. Compared to the concentration of photogenerated carriers, the number of charges transferred between gas molecules and the sensing material becomes less significant. Thus, the conductivity changes as well as the gas sensitivity are reduced under illumination [84, 86]. Wu et al. observed that the O_3_ gas response of PbS QDs is decreased under 450–650 nm LED and fluorescent lamps [84]. It is to note that the gas sensitivity of 2D metal chalcogenides, such as MoS₂ nanosheets toward NO_2_, is enhanced under illumination. In this case, the incident photons are expected to mainly facilitate the desorption of O₂⁻, thereby exposing more active sites to NO_2_ adsorption and direct charge transfer [27].

#### State-of-the-art metal chalcogenide QD gas sensors

The tunable optical and electrical properties, endowed by advancements in synthesis and post-synthesis treatments, allow metal chalcogenide QDs to show desirable gas sensing characteristics at room temperature. The state-of-the-art performance of metal chalcogenide QD gas sensors is summarized in Table 1.

**CHEMICAL AND MECHANICAL STABILITY.** Chemical poisoning of metal chalcogenide QDs from the environment, like O_2_ and H_2_O, can gradually deteriorate the sensitivity of the gas sensor. To improve the chemical stability, Liu et al. developed PbSe QD gas sensors by cation exchange and maintained 85.2% of the response after 20 days [88]. Surface passivation using halogen ligand treatment is another effective strategy, which has been described in Sect. 3.1.2. Metal chalcogenide QDs are readily integrated on flexible and porous substrates through solution-based fabrication techniques, and the sensor devices demonstrated mechanical stability. For example, a PbS QD-based gas sensor has been made on a paper substrate, which exhibited only a 7.1% decrease in gas response after 5000 bending cycles, as shown in Fig. 4h [19]. Zeng et al. proposed a fully stretchable gas sensor made of a PbS QD layer and an elastomer film (VHB acrylic 4910) that demonstrated negligible changes in response to 50 ppm NO_2_ even after 1000 mechanical bending cycles [89].

**Table 1:** State-of-the-art performance of metal chalcogenide QD gas sensors

Materials	Mechanism	Temp.	Target gases	Gas Conc. [ppm]	Gas response	LOD	T_90_/ T_10_ [s]	Ref.
PbS QDs	Chemiresistive	RT	NO_2_	50	21.7	84 ppb	12/37	[19]
PbS QDs (Spray-coated)	Chemiresistive	RT	NO_2_	50	44	56 ppb	1/28	[81]
PbS QDs (stretchable)	Chemiresistive	RT	NO_2_	50	125.0	13 ppb	7/22	[89]
PbS QDs	Chemiresistive	RT	NO_2_	50	55	NA	6/28	[77]
PbS QDs	Chemiresistive	RT	NO_2_	12.5	~ 8.5	0.78 ppb	30/2700	[29]
PbS QDs	Chemiresistive	RT	NO_2_	10	7.9	NA	79/73	[79]
PbS QDs (photoexcited)	Chemiresistive	RT	O_3_	0.12	2.4	1.34 ppb	160/210	[84]
PbSe QDs	Chemiresistive	RT	NO_2_	50	22.3	NA	7/39	[88]
PbSe QDs (photoexcited)	Chemiresistive	RT	NO_2_	1.32	~ 50%^a^	3 ppb	27/102	[86]
CdS QD gel	Chemiresistive	RT	NO_2_	1.79	~ 17%^a^	11 ppb	29/28	[82]
Pb_0.09_Cd_0.91_Se QDs	Chemiresistive	RT	NO_2_	1.32	1300%^a^	3 ppb	31/63	[80]
PbS QDs	Optical	RT	NH_3_	1000	0.72 nA^b^	481 ppm	NA	[90]
PbS QDs	Optical	RT	NO_2_	1	35 pm^c^	1 ppb	NA	[91]
CdSe QDs	Optical	RT	toluene	750	1.1^d^	50 ppm	NA	[92]
			xylene		2.2^d^	15 ppm	NA	

^a^: The gas response is calculated by the equation $\:\frac{|{R}_{a}-{R}_{g}|}{{R}_{a}}.$^b^: The change of photocurrent after exposure to NH_3_. ^c^: Shift of adsorption spectrum owing to gas adsorption-induced refraction index change (pm: picometer). ^d^: PL intensity change

### 1D metal chalcogenides − nanorods and nanowires

One-dimensional (1D) metal chalcogenides, including nanowires (NWs) and nanorods (NRs), are strongly confined in the radial dimension but elongated in length. One of the key advantages of assembling 1D-based networks is to enhance gas adsorption and diffusion, while the continuous conductive pathways enable efficient charge transport and mechanical flexibility. Recent advancements in 1D nanostructure engineering have further enhanced room-temperature gas sensing performance [93, 94].

Unlike QDs with hopping charge transport across nanoparticle arrays, 1D NWs provide directional electron transport channels, reducing interfacial resistance. The porous microstructure networks further allow gas molecules to diffuse efficiently into active sensing regions, enhancing detection sensitivity and response speed [95]. A room-temperature NO_2_ sensor based on PbS NWs leveraged these advantages, exhibiting a response of 17.5 toward 50 ppm NO_2_, with a detection limit of 36 ppb and a rapid response time of 3 s [96]. Furthermore, the highly interconnected conductive network of 1D PbS nanostructures mitigated mechanical strain-induced conductivity loss and retained 94% of its initial response after 500 bending cycles, whereas QD-based sensors suffered 15% degradation under similar conditions.

Another 1D nanostructure-based approach for room-temperature NO_2_ sensing was demonstrated using CdS NWs, where a light-activated mechanism was employed to enhance gas detection efficiency [94]. The sensor exhibited a 236% response to 10 ppm NO_2_ and an 11% response to 12.5 ppb NO_2_, achieving a detection limit of 12.5 ppb. The incorporation of green-light activation facilitated photoinduced charge separation, accelerating adsorption–desorption kinetics and enabling ultrafast response and recovery time. This synergistic effect of sulfur vacancy engineering and photo-carrier generation overcomes the sensitivity and selectivity limitations of conventional room-temperature NO_2_ sensors.

Suh et al. developed a high-performance NO_2_ gas sensor utilizing 1D WS_2_ nanostructures [97]. The study involved the growth of WS_2_ on highly porous SiO_2_ NR templates, enabling the formation of edge-exposed 1D WS_2_ flakes with numerous active sites. The WS_2_ NR-based sensor exhibited a gas response of 151.2% toward 5 ppm NO_2_ at room temperature, with a theoretical detection limit as low as 13.7 ppb. The superior sensing performance is attributed to the highly porous 1D nanostructure and the high density of reactive edge sites, which serve as favorable adsorption sites for target gas molecules.

### 2D metal chalcogenides − nanosheets

**Fig. 5:**
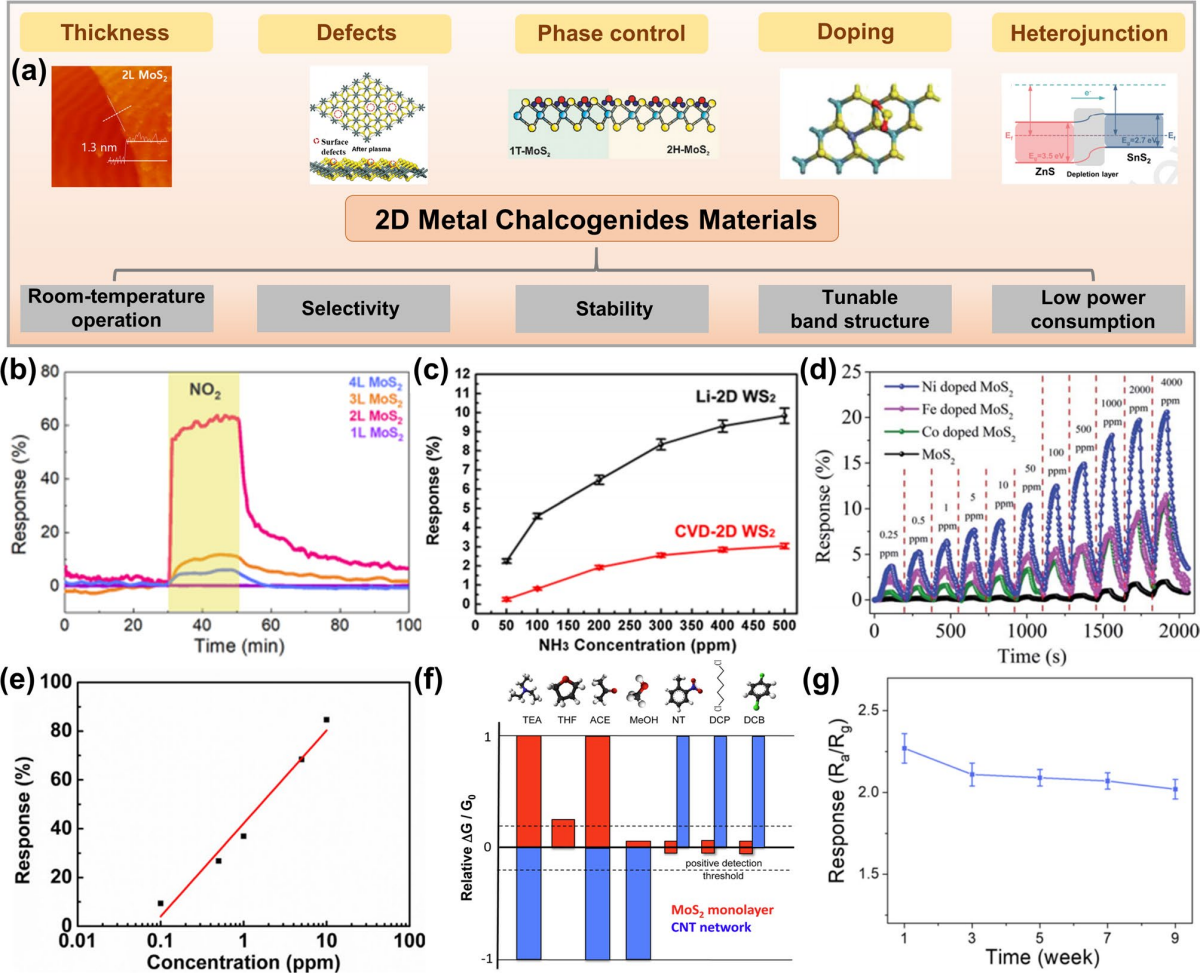
2D metal chalcogenides for gas sensing. (**a**) Performance metrics and design strategies for 2D metal chalcogenide-based gas sensors. (**b**) Thickness-dependent gas response of 2D MoS_2_ towards NO_2_ [109]. (**c**) Comparison of NH_3_ response between 2D WS_2_ synthesized by lithium-ion intercalation-assisted chemical exfoliation (black) and by CVD (red) [110]. (**d**) Dynamic SO_2_ response based on 2D MoS_2_ gas sensors doped with different elements [113]. (**e**) Logarithmic relationship between gas response and NO_2_ concentration for WS_2_ nanosheet gas sensors [117]. (**f**) Response of 2D MoS_2_ sensors selectively for various gases [120]. (**g**) Stability of WSe_2_ gas sensors over time [124]

Two-dimensional (2D) metal chalcogenides have emerged as another promising material platform for wearable gas sensors due to the unique combination of their high surface area, chemical reactivity, and electrical properties. Their exceptional sensing properties and materials design versatility offer advantages such as room-temperature operation, high selectivity and stability, tunable band structures, and low power consumption, which can be controlled through various strategies (Fig. 5a) [24, 98].

#### Synthesis of 2D metal chalcogenides

2D metal chalcogenides can be synthesized using a variety of methods, including chemical vapor deposition (CVD) [99], chemical and mechanical exfoliation [100], atomic layer deposition (ALD) [101], and colloidal synthesis [102]. Among these, CVD and chemical exfoliation are most widely employed in the fabrication of 2D metal chalcogenide-based gas sensors, as they can produce high-quality and scalable layered materials with precisely tunable physical properties [103].

The CVD process involves introducing gaseous precursors into a reaction chamber, where they are thermally decomposed or react at the surface of a heated substrate to form solid products [104]. These precursors undergo heterogeneous reactions at the solid-gas interface, resulting in the nucleation and epitaxial growth of thin films. Critical parameters such as reaction temperature, pressure, precursor flow rates, and substrate type must be precisely controlled to ensure high crystallinity, layer uniformity, and compositional consistency. Following film deposition, post-processing steps such as wet etching or polymer-assisted transfer are typically performed to relocate the 2D films onto target substrates. The CVD technique supports wafer-scale synthesis, enables atomic-level thickness control, and allows for in situ doping and heterostructure integration [105]. Its compatibility with current semiconductor process flows further facilitates the direct synthesis of 2D materials on device substrates, thereby streamlining sensor fabrication workflows [103]. However, the need for high-temperature furnaces, toxic or unstable precursors, and stringent control over reaction environments presents challenges for widespread adoption and limits the versatility of CVD for certain material systems.

Chemical exfoliation is a top-down synthesis approach that produces 2D flakes by chemically disrupting the interlayer van der Waals interactions in bulk layered crystals. Typically, the bulk precursor is exposed to reactive chemical agents, such as lithium intercalants or strong acids, that intercalate between layers, weakening interlayer bonding and enabling subsequent exfoliation via ultrasonication or solvent dispersion [106]. This method allows for scalable and low-cost production of 2D materials, such as MoS_2_, and is particularly attractive for industrial-scale applications due to its high throughput and ambient-condition operation. Liquid-phase exfoliation yields monolayer or few-layer flakes with a thickness typically reduced to a median size of ~ 80 nm after processing. Notably, the exfoliation process introduces intrinsic sulfur vacancies on the basal planes, which serve as active adsorption sites for gas molecules and can be further engineered through substitutional doping (e.g., nitrogen incorporation via N-methyl pyrrolidone degradation) to tailor the material’s gas-sensing selectivity and sensitivity [107]. Moreover, the room-temperature operation mitigates risks associated with high-temperature degradation, such as oxidation or structural distortion [108]. Despite these advantages, chemical exfoliation suffers from several limitations, including the difficulty in precisely tuning vacancy densities and spatial distributions, degradation of flake morphology during processing, and the necessity for rigorous reaction condition optimization. These factors, combined with the long recovery times observed in some sensing applications, can negatively impact sensor reproducibility and stability [108].

#### Design strategies for 2D metal chalcogenides

The adjustable surface sites and energy band structures of 2D metal chalcogenides offer a versatile platform for optimizing gas sensing performance. Modifications of the layer thickness, defect engineering, phase control, doping, heterojunction formation, and other strategies allow for precise tuning of the bandgap and electronic states, thereby enhancing gas-surface interactions, charge transfer rates, and sensing selectivity.

**THICKNESS**. The Schottky barrier height between 2D metal chalcogenides and metal contacts is thickness-dependent, which can be modulated to enhance the gas sensitivity. When varying the thickness of CVD-grown 2D MoS_2_ from monolayer to four layers, the bilayer MoS_2_ configuration exhibited the highest sensitivity toward NO_2_ (Fig. 5b) [109]. It is attributed to an optimized Schottky barrier height at the metal-semiconductor interface. This highlights the effectiveness of electronic band alignment engineering via layer control in improving charge transfer rates. Moreover, the CVD process provides uniform, large-area synthesis of thin layers, supporting practical applications in scalable device fabrication.

**DEFECT ENGINEERING**. Introducing surface defects has also been shown to enhance gas adsorption through local active sites. In one approach, few-layer WS_2_ nanosheets with a high density of sulfur vacancies (V_S_) were synthesized via lithium-ion intercalation-assisted chemical exfoliation (Fig. 5c) [110]. The resulting material exhibited significantly improved sensitivity to NH_3_ compared to highly crystalline CVD-grown WS_2_, due to the abundance of defect-induced adsorption sites. Dai et al. developed Ar plasma irradiation to produce V_S_ and modulate the electronic structure of SnS_2_. The plasma-treated SnS_2_ exhibited high sensitivity and selectivity toward NH_3_ owing to highly active V_S_ on the surface. However, high surface reactivity also makes the system sensitive to air interference, like O_2_ and H_2_O exposure, leading to poor long-term stability [111].

**PHASE CONTROL.** Different crystalline phases in 2D metal chalcogenides can exhibit different interactions with target gas molecules. 2D MoS_2_ with coexisting 1T and 2 H phases, synthesized via in-situ ethylene glycol intercalation, exhibited superior detection capability for toluene at room temperature [112]. The metallic 1T phase contributed a better electron transport due to enhanced conductivity and high sensitivity due to strong gas adsorption energies, while the semiconducting 2 H phase provided structural stability. Compared to pure 2 H-MoS_2_, the mixed-phase structure enabled both higher response and faster recovery, highlighting the functional synergy between crystal phases.

**DOPING.** Doping of 2D metal chalcogenides with metal atoms has proven effective in tuning the electronic structure and enhancing surface-gas interaction. Hydrothermally synthesized Ni-, Fe-, and Co-doped MoS_2_ films revealed that doping significantly improved SO_2_ sensitivity [113]. The enhanced response was attributed to stronger adsorption energy between the doped surface and SO_2_ molecules, which promoted charge transfer rate and resistance change. Comparatively, the Ni-doped samples show the best responses, the Fe- and Co-doped samples show moderate responses, and the pristine MoS_2_ exhibits the lowest sensitivity (Fig. 5d). Cation doping can induce the anion vacancies in the meantime. Yang et al. developed In^2+^-doped SnSe_2_ with a high sensitivity of 4.85 ppm^− 1^, a LOD of 3.46 ppb, and short reaction time toward SO_2_. Although substituting the Sn with In atoms can reduce the adsorption energy of SO_2_, DFT calculations indicate that the cation doping-induced Se vacancies significantly enhance the gas response [30].

**HETEROJUNCTION FORMATION.** Construction of heterojunctions is an efficient strategy to modulate the surface properties and electronic structures by stacking two or more 2D metal chalcogenides into multi-layer materials. Taking the binary component as an example, the type of heterojunction can be divided into n-n, p-p, and n-p junctions. The formation of type-II heterojunctions has enabled further improvement in charge carrier dynamics. ZnS/SnS_2_ heterostructure nanoflakes, synthesized via hydrothermal methods, demonstrated a high response value of 140 to 10 ppm NO_2_ under UV irradiation [114]. The heterojunction facilitated efficient charge separation and suppressed electron-hole recombination by providing spatially separated transport pathways, thereby improving both sensitivity and detection stability under light-assisted conditions. Wu et al. synthesized the 2D/2D ZnS_2_/SnS_2_ van der Waals heterojunction by physical mixing the precursors in hydrothermal growth. This heterojunction exhibited improved gas response, which can be further enhanced with the increasing amount of heterointerfaces. DFT and XPS analysis revealed that the boosted gas performance is due to improved charge transfer rate at the interface and strong NO_2_ adsorption energy [115].

#### State-of-the-art 2D metal chalcogenide gas sensors

**ROOM-TEMPERATURE OPERATION**. The presence of surface chemically active sites, including dangling bonds and defects, and the high specific surface area facilitate gas adsorption on 2D metal chalcogenides, enabling high charge transfer rates [116]. As a result, 2D metal chalcogenide-based gas sensors exhibit high sensitivity even without external heating [22]. For example, ultra-thin WS₂ nanosheets have been utilized to develop a high-performance NO_2_ sensor operated at room temperature [117]. The sensor exhibited a logarithmic relationship between response and NO_2_ concentration within the tested range (Fig. 5e), covering low concentrations relevant to environmental monitoring and safety applications. Similarly, single-layer MoSe_2_ has been explored as a room-temperature gas sensor to detect NH_3_ at as low as 50 ppm, leveraging the high surface-to-volume ratio and tunable electronic properties of 2D transition metal dichalcogenides (TMDCs) [118]. The sensitivity increases proportionally with concentration, demonstrating its potential for high-sensitivity gas detection. Beyond TMDCs, cross-linked p-type SnS nanoplates on SiO_2_ nanorods have been developed as a room-temperature NO_2_ sensor, exhibiting high sensitivity with a 116% response to 5 ppm NO_2_ and an ultra-low detection limit of 21 ppt [119].

**SELECTIVITY.** 2D metal chalcogenides outperform traditional materials, such as metal oxides and carbon nanotubes (CNTs), in selectively sensing various organic vapors [120]. This advantage is mainly attributed to the adaptable interaction with different gas molecules [24]. For instance, Perkins et al. demonstrated a monolayer MoS₂ gas sensor capable of detecting a range of VOCs, including dichlorobenzene, triethylamine (TEA), nitrotoluene, nitromethane, dichloropentane, and water [120] (Fig. 5f). The sensor exhibited a strong, concentration-dependent response to TEA, a decomposition marker of V-series nerve agents, with conductivity changes (ΔG/G₀) increasing across a wide range (0.002% P₀ to 0.2% P₀). Importantly, the MoS₂ sensor showed minimal response to water vapor, demonstrating high specificity for electron donor species in complex environments.

**CHEMICAL AND MECHANICAL STABILITY.** 2D metal chalcogenide gas sensors operate more stably at room temperature than those based on graphene, MXenes, and black phosphorus, which often suffer from degradation under exposure to oxygen, moisture, and light illumination [121–123]. For example, Zhang et al. reported a WSe_2_ gas sensor synthesized via liquid-phase exfoliation, which exhibited no significant performance degradation in response to 1 ppm NO_2_ over 9 weeks of continuous testing (Fig. 5g) [124]. The sensor also maintained functionality under varying relative humidity levels (30–90%), with only a slight decrease in response attributed to competition between water molecules and NO_2_ for adsorption sites. In another example, Bi_2_Se_3_, a topological insulator, benefits from topological surface states (TSSs) derived from the non-trivial topology of its bulk bands. These TSSs are inherently robust against surface defects and impurities, thereby improving sensor stability in chemically complex environments [125]. 2D metal chalcogenides also exhibit excellent mechanical stability, a critical requirement for wearable applications. Kim et al. developed a flexible CO gas sensor by integrating Au-functionalized WS_2_ nanoflakes onto a polyamide substrate, demonstrating resilience under repeated mechanical stress [126]. After 1000 torsion cycles, the sensor’s response to 1, 10, and 50 ppm CO decreased by only 4.1%, 6.9%, and 8.1%, respectively. Even when bent to a curvature radius of 4 mm, the sensor maintained nearly unchanged performance, with less than 1% reduction in response at all tested concentrations.

**LOW POWER CONSUMPTION.** The atomically thin structure of 2D metal chalcogenides enables efficient charge transport rates over short distances, reducing the power consumed by external heating elements or large driving voltages, which are typically required by bulk or metal oxide-based sensors [127, 128]. For example, a gas sensor based on a large-area WS₂ₓSe₂₋₂ₓ alloy demonstrated power consumption as low as 0.75–21 µW during operation at room temperature [129]. In addition, the low-voltage operation of 2D chalcogenide devices reduces the risk of overheating and deterioration of flexible or stretchable substrates in wearable sensors.

### Nanocomposites−low-dimensional metal chalcogenides and other nanomaterial hybrids

The sensing properties of a single material component may be limited by the trade-off between recovery speed, humidity interference, and chemical stability, and other metrics. Therefore, integration of multiple low-dimensional materials can form synergistic effects and enhance gas sensing performance compared to the individual constituents. An effective approach is to hybridize metal chalcogenides with other nanomaterials, such as graphene, MXenes, and metal oxides. Such heterostructures combine the transducing effect of metal dichalcogenides originating from surface reactivity and tunable electronic properties with the high electrical conductivity, mechanical flexibility, and chemical functionality of the support materials [98]. These nanocomposites can be meticulously designed to enhance charge transfer rates, engineer active surface sites, and optimize interfacial interactions, simultaneously improving sensitivity, selectivity, stability, and response dynamics.

A representative example of the metal/semiconductor heterostructure involves a graphene/MoS_2_ film, developed for NO_2_ detection under ambient conditions [130]. In this configuration, the MoS_2_ phase contributes a high surface-to-volume ratio and gas adsorption capability, while graphene ensures excellent electrical conductivity and rapid charge transport rates. The resulting nanocomposite sensor exhibited a negative resistance response to NO_2_ and faster recovery time, along with outstanding mechanical durability, maintaining performance after 5000 bending cycles and long-term stability over 19 months. Jiao et al. synthesized SnS_2_ QD/graphene nanocomposites with the semiconducting type tunable by adjusting the component proportions [85]. The relative variation of the charge carrier density of the nanocomposite is much larger than that of the pristine graphene, resulting in higher conductivity changes and gas sensitivity. The sensor based on the SnS_2_ QD/graphene realized room-temperature detection toward 125 ppb of NO_2_ with 860% response, 114 s of response time, and 166 s of recovery. Light with different wavelengths and intensities was proposed to enhance gas sensitivity owing to more surface adsorption sites exposed under illumination. For example, red light (1 mW/cm^2^) greatly enhances the sensitivity of the SnS_2_ QD/graphene gas sensor up to 5.1-fold. A different approach utilized Ti_3_C_2_T_x_ MXene/WS_2_ heterostructures fabricated on flexible paper-based substrates for NO_2_ sensing [131]. The combination of high conductivity and surface functional groups from MXene with the reactive surface and semiconducting behavior of WS_2_ enabled detection of NO_2_ at room temperature, with a response of 15.2% to 1 ppm NO_2_, and a detection limit as low as 11 ppb. The sensor demonstrated strong humidity tolerance, maintaining functionality at 90% relative humidity, and retained stable performance after 500 mechanical bends and 40 days of continuous operation, with only 24.3% decay in response.

Enhancement in sensing performance has also been realized through p-n heterojunction formation. A MoS_2_/ZnO nanocomposite, formed by decorating 2D MoS_2_ nanosheets with ZnO nanoparticles, created a p-n junction that promotes electron-hole separation and reduces recombination losses, thereby improving response speed and sensitivity [132]. The strong interfacial interaction also facilitated gas adsorption and desorption kinetics. The optimized sensor achieved a remarkable response value of 3050% to 5 ppm NO_2_, which is 11 times higher than that of pristine MoS_2_, and retained excellent stability over 10 weeks of operation.

## Wearable device integration

The tunable physical properties and efficient transducing effects make low-dimensional metal chalcogenides promising materials for high-performance gas sensors with low-power consumption. Building upon these devices, a truly wearable gas-sensing system is under the way by further solving the power and data transmission challenges. Such integrated sensor array systems will be augmented by AI algorithms to identify complex gas mixtures and recognize different odors.

### Wireless sensory data communication

**Fig. 6:**
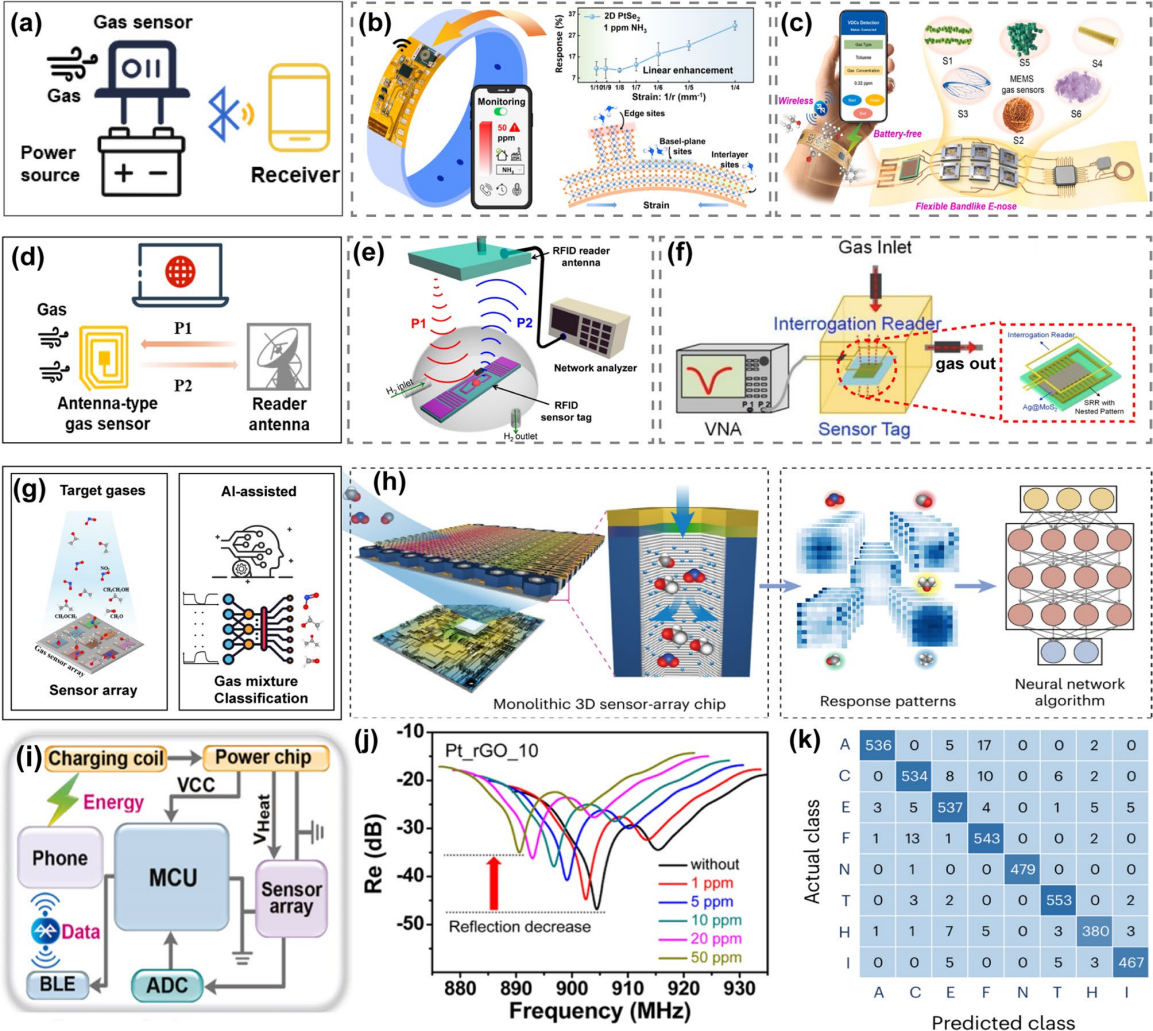
Wearable gas sensing device integration. (**a**) Schematics of a gas sensor system with power sources and wireless data transmission modules. (**b**) Device schematics, sensing mechanisms, and performance of a flexible NH_3_ gas sensing belt based on 2D PtSe_2_ under 1 ppm NH_3_ exposure [133]. (**c**) Illustration of the wirelessly charged and flexible e-nose system based on metal-oxide MEMS gas sensors [135]. (**d**) Schematics of passive antenna-type gas sensors. (**e**) Schematics of the ultrahigh frequency (UHF)-RFID-based wireless gas sensor system composed of an RFID sensor tag and RFID-antenna-connected network analyzer [136]. (**f**) Measurement of a wireless NH_3_ detector containing an Ag@MoS_2_-coated SRR sensor tag and interrogation reader [137]. (**g**) Schematics of AI-empowered gas sensor arrays. (**h**) Structures of a monolithic biomimetic olfactory chip with the integrated circuit (left) and its response patterns for different odour/gas molecules and the algorithm design for pattern recognition (right) [139]. (**i**) The schematic circuit diagram of the wirelessly charged e-nose device shown in (c) [135]. (**j**) Change in the RF relectance properties of different Pt_rGOs-based wireless sensors as a function of the hydrogen gas concentration measured at a distance of 10 cm [136]. (**k**) Confusion matrix of the actual class and predicted class when recognizing eight gases: acetone (A), carbon monoxide (C), ethanol (E), formaldehyde (F), nitrogen dioxide (N), toluene (T), hydrogen (H), and isobutylene (I) [139]

**WIRELESS MODULE INTEGRATION**. To further improve wearability and data visualization, wireless transmission of sensory data is desirable. Many of the low-powered gas sensors have been readily integrated with data acquisition units and wireless communication units to form circuits powered by portable batteries (Fig. 6a). The analog data collected from the sensors is converted to digital signals and transmitted wirelessly to mobile terminals for further processing and display. Tao et al. demonstrated such wearable platforms by assembling a PtSe_2_ gas sensor, a data acquisition circuit, and a Bluetooth Low Energy (BLE) module on a flexible printed circuit board (Fig. 6b) [133]. The sensor response is displayed on a computer in real time, which is not altered by the BLE interconnects. Other wireless technologies can also be employed based on the requirements of transmission rate, distance, and power consumption. For example, gas sensors connected to a Zigbee wireless network cover a longer communication distance with lower energy but can only transmit at a lower data rate [134]. Instead of using batteries, wireless power supplies have been utilized to support low-power gas sensors. As shown in Fig. 6c and i, a wearable metal oxide semiconductor sensor system can be remotely charged by cell phones through the charging coil [135]. The intermittent power input is managed by a power chip before supplying voltages to other components in the circuit. The sensory data is transmitted to the cell phone through a BLE module. Therefore, mobile devices like smartphones become both a power source and data receiver, actively interrogating the wearable sensor system.

**ANTENNA-TYPE GAS SENSORS**. Radio frequency identification (RFID) technologies have been developed as crucial enablers for Internet of Things (IoT) sensor networks, thanks to their simple and compact device structure, as well as their passive and power-free operation. In the RFID tag, resonators, such as antennas, strongly scatter incident electromagnetic waves at certain resonance RFs. The reflection spectra are modulated in wavelength and magnitude when the resonator’s physical properties are changed (Fig. 6d). Gas-sensitive materials can be integrated with the RF antenna, such that the transduced sensory data is embedded in the reflected signal of the RFID tag upon being queried by an RFID reader. Jang et al. coated Pt-decorated reduced graphene oxide (rGO) on part of a commercial flexible RFID tag antenna (Fig. 6e) [136]. As the H_2_ gas concentration increases, the resistance of Pt-rGO increases, leading to impedance mismatching between the antenna and IC chip. Correspondingly, the reflection spectra of the RFID tag are also decreased in magnitude and shifted in phase **(**Fig. 6j**)**. The adsorption of gas molecules can also cause changes in the dielectric constant of the sensing material layer, which determines the resonance of RF sensors. Chen et al. fabricated split-ring resonators (SRRs) immobilized with Ag nanoparticles-decorated MoS_2_ nanosheets (Ag@MoS_2_) (Fig. 6f) [137]. When the tag is exposed to NH_3_, the dielectric constant of Ag@MoS_2_ is increased, and the effective capacitance of the SSR is higher. The resultant shift of the resonance frequency is recorded by a network analyzer and correlated with the NH_3_ concentration.

### AI-empowered gas sensor arrays

The gas sensing environment is typically complex, where different types of gas molecules with a wide range of concentrations co-exist. Human breath primarily consists of N_2_, O_2_, CO_2_, H_2_, and H_2_O, and also contains trace amounts of NO_2_, NO, NH_3_, CO, H_2_S, along with up to 3500 different kinds of VOCs [138]. Some of these volatile molecules, although below ppm levels, are correlated with diseases and health status. Therefore, high sensitivity as well as selectivity are critical metrics of wearable gas sensors for VOCs gas mixture classification and odor recognition.

The performance of a single gas sensor is essentially limited by the surface chemical-physical process. The output of a single sensor is one-dimensional with limited features, making it insufficient to support comprehensive data analysis algorithms. Instead, the collaboration between gas sensor arrays and machine learning algorithms has been proposed to greatly enhance the sensing functionality and promise artificial olfactory systems (Fig. 6g). Gas sensors with different gas responses can be utilized to generate multidimensional data, such as image patterns, under single or multiple gas exposures. These data matrices are labeled to train machine learning algorithms for gas type classification and concentration prediction. To build arrays of distinguishable gas sensors, an efficient approach is to precisely vary the composition of the sensing materials across different sensors. Fan et al. fabricated a large-scale sensor array by introducing a multicomponent interfacial layer (MCI) on a porous alumina membrane, i.e., depositing ZnO, NiO, In_2_O_3_, and WO_3_ with composition gradients on nanotubular PdO/SnO_2_ arrays (Fig. 6h) [139]. After convolving with the basic response of the nanotube array, the MCI layer with well-controlled elemental distribution can offer a wide range of gas responses across the sensor array. Therefore, distinctive 2D response patterns are obtained corresponding to specific gas types and concentrations, where each pixel value is the normalized output signal of an individual sensor node. A large number of response patterns are used to train convolutional neural networks (CNNs) for high-accuracy single and multiple-gas classification **(**Fig. 6k**)**. To improve computation efficiency, the gas response pattern has been processed first by principal component analysis (PCA) and fed to multi-layer CNN models. Based on a similar device array mechanism, other studies also suggest that visualizing all features of a gas sensing cycle, such as the dynamic response and recovery curve, in 2D patterns can provide more diversified and high-quality training data for AI algorithms [140]. The increased data dimensionality necessitates more advanced deep learning algorithms, which can reduce the number of sensors needed to achieve the same level of performance [141].

## Conclusions and outlook

Low-dimensional metal chalcogenides have shown unique advantages in achieving low-power wearable gas-sensing devices. This review summarizes recent progress related to low-dimensional metal chalcogenide gas sensors, including suitable transducing mechanisms, the synthesis and material design strategies of four low-dimensional metal chalcogenide systems, and their device integration as wearable sensing systems. Their high-performance gas sensing capabilities at room temperature are fundamentally enabled by appropriate surface adsorption energies and charge transfer rates, which can be fine-tuned during synthesis and/or through post-synthesis treatment. These material design strategies are also compatible with sensor array integration, providing versatile routes to achieve low-power operation, wireless data transmission, and multi-gas classification. However, despite great breakthroughs made in previous research, there are still some challenges for state-of-the-art low-dimensional metal chalcogenide gas sensors.

**SENSING MECHANISMS**. The existing mechanistic explanations for metal chalcogenide gas sensors are not well-established, some of which are even contradictory, limiting further rational material design. Surface adsorption energies have been investigated using various surface probing techniques and DFT simulations. However, how NO_2_ molecules are bound to the surface sites of PbS QDs is still unclear. Some literature proposed that NO_2_ adsorbs directly on the surface site and competes with O_2_, while other studies suggest that NO_2_ is sensed through pre-adsorbed O_2_^−^ species [29, 77]. As discussed in Sect. 2.1, comprehensive studies on all the influential factors are required to determine the dominant gas-sensing mechanism. Secondly, it is lacking a holistic theoretical model to illustrate how materials’ physical parameters, including the band structure, doping concentration, carrier mobility, and carrier lifetime, interplay to determine gas sensing performance. One parameter change may induce complex and intertwined alterations in the gas adsorption and charge transfer processes. Also, external stimuli other than voltages, such as light illumination, have been introduced to modulate the gas sensing performance. As mentioned previously, light-illuminated metal chalcogenide QDs and nanosheets can show the opposite trend in overall sensitivity, possibly due to different photocarrier generation efficiencies. A more detailed investigation should be carried out to understand how different photon energies facilitate gas desorption and adsorption and simultaneously alter the carrier transfer process. Therefore, more effective in-situ characterization techniques for the gas sensing process would be valuable to elucidate these mechanisms directly. For instance, in situ diffuse reflectance infrared Fourier transform spectroscopy (DRIFTS) was applied to measure the film resistance and surface Fourier transform infrared spectroscopy (FTIR) simultaneously, proving that NO_2_ attracts electrons from PbS QDs as an effective “p-type donor” [29]. In situ X-ray photoelectron spectroscopy (XPS) characterization was developed to observe the partial desorption of oxygen species on the SnS_2_/TiO_2_ surface due to the illumination by a 525 nm light source [142].

**PERFORMANCE METRICS**. Despite a variety of strategies from synthesis to post-treatment reviewed in this paper, the gas sensing performance of low-dimensional chalcogenides still has a large room for improvement in sensitivity, response/recovery time, selectivity, and stability. Higher sensitivity for low-concentration gases is still needed. At least ppb-level LOD is required to detect biomarker gases in exhalation for health monitoring applications. Mixed components in exhaled gases also complicate analysis of the target gases if the sensitivity and selectivity are not sufficiently high. Another challenge is to balance the sensitivity and response/recovery time. While increasing the surface adsorption energy for target gases can enhance sensitivity, it may also make the desorption process more difficult, thereby hindering rapid recovery. The long-term stability of the sensor needs further development for practical applications. The highly reactive metal chalcogenide surface is prone to binding with water molecules, and thus, the device stability can be rapidly compromised under high humidity. Such material oxidation and degradation can be even accelerated under light illumination. For improving the sensing performance, one potential approach is to construct 3D porous nanostructures for more exposed active sites and better gas molecule diffusion. Compared to other material systems, low-dimensional metal chalcogenides can leverage flexible post-assembly methods. For example, ink printing technologies and lithographic techniques have been utilized to assemble 3D metal chalcogenide structures [143, 144].

**MULTIDIMENTIONAL DATA**. As an ultimate goal, wearable gas sensing systems will accurately distinguish and detect target gases from complex gas environments. It is promising to form gas sensor arrays and use the sensory data matrix to train machine learning algorithms, but the classification accuracy depends on the diversity and quantity of the collected data. Several examples have successfully integrated different sensors with varied compositions and gas selectivity into one sensor array. However, it is still challenging for most gas-sensing material systems, including metal chalcogenides, to guarantee consistency of such composition distribution in sensor arrays from sample to sample and during scaling up. Therefore, the training data acquired from different sensor arrays may be less reliable. A possible alternative approach is to obtain multi-dimensional data from the same sensor array by applying different external stimuli. External fields can modulate the gas response of low-dimensional metal chalcogenides through various mechanisms to further diversify the output data. Also, multi-variable gas sensor structures can be designed to transduce the gas concentration to more than one type of independent output signals, including current, potential, capacitance, and optical spectral features, to create multi-dimensional data matrices [145].

## Data Availability

Not applicable.
